# Protective Effect of Polydatin on Jejunal Mucosal Integrity, Redox Status, Inflammatory Response, and Mitochondrial Function in Intrauterine Growth-Retarded Weanling Piglets

**DOI:** 10.1155/2020/7178123

**Published:** 2020-10-10

**Authors:** Hao Zhang, Yanan Chen, Yue Li, Tian Wang

**Affiliations:** ^1^College of Animal Science & Technology, Nanjing Agricultural University, Nanjing 210095, China; ^2^Postdoctoral Research Station of Clinical Veterinary Medicine, College of Veterinary Medicine, Nanjing Agricultural University, Nanjing, Jiangsu, China 210095; ^3^Shanghai Key Laboratory of Veterinary Biotechnology, Shanghai 200240, China; ^4^Institute of Animal Science, Jiangsu Academy of Agricultural Sciences, Nanjing 210014, China

## Abstract

Intrauterine growth retardation (IUGR) delays the gut development of neonates, but effective treatment strategies are still limited. This study used newborn piglets as a model to evaluate the protective effect of polydatin (PD) against IUGR-induced intestinal injury. In total, 36 IUGR piglets and an equal number of normal birth weight (NBW) littermates were fed either a basal diet or a PD-supplemented diet from 21 to 35 days of age. Compared with NBW, IUGR induced jejunal damage and barrier dysfunction of piglets, as indicated by observable bacterial translocation, enhanced apoptosis, oxidative and immunological damage, and mitochondrial dysfunction. PD treatment decreased bacterial translocation and inhibited the IUGR-induced increases in circulating diamine oxidase activity (*P* = 0.039) and D-lactate content (*P* = 0.004). The apoptotic rate (*P* = 0.024) was reduced by 35.2% in the PD-treated piglets, along with increases in villus height (*P* = 0.033) and in ratio of villus height to crypt depth (*P* = 0.049). PD treatment promoted superoxide dismutase (*P* = 0.026) and glutathione S-transferase activities (*P* = 0.006) and reduced malondialdehyde (*P* = 0.015) and 8-hydroxy-2′-deoxyguanosine accumulation (*P* = 0.034) in the jejunum. The PD-treated IUGR piglets showed decreased jejunal myeloperoxidase activity (*P* = 0.029) and tumor necrosis factor alpha content (*P* = 0.035) than those received a basal diet. PD stimulated nuclear sirtuin 1 (*P* = 0.028) and mitochondrial citrate synthase activities (*P* = 0.020) and facilitated adenosine triphosphate production (*P* = 0.009) in the jejunum of piglets. Furthermore, PD reversed the IUGR-induced declines in mitochondrial DNA content (*P* = 0.048), the phosphorylation of adenosine monophosphate-activated protein kinase alpha (*P* = 0.027), and proliferation-activated receptor gamma coactivator 1 alpha expression (*P* = 0.033). Altogether, the results indicate that PD may improve jejunal integrity, mitigate mucosal oxidative and immunological damage, and facilitate mitochondrial function in IUGR piglets.

## 1. Introduction

Intrauterine growth retardation (IUGR) refers to the failure of a fetus to achieve its genetic growth potential. This clinical problem affects approximately 5–10% of pregnancies and remains a predisposing factor for perinatal morbidity and mortality [1]. In most cases, IUGR results from uteroplacental insufficiency where a hemodynamic adaptation occurs with blood flow redistribution in the fetal body. This redistribution favors adequate perfusion to the vital organs like the brain and the heart to support the survival of fetus under adverse intrauterine circumstances, but it inevitably limits the supply of oxygen and nutrients to other organs including the small intestine [2]. Consequently, IUGR can delay the normal development of the small intestine in neonates [3–6].

The small intestine is the key location for nutrient digestion and absorption, but it also serves as a critical barrier against various infectious agents [7]. Previous studies have demonstrated that IUGR impairs small intestinal morphology, disturbs redox and immune status, and induces enterocyte apoptosis and mucosal damage. The end result is a decrease in the ability of neonates to resist diseases and, ultimately, a compromised growth performance [4–6, 8, 9]. Finding appropriate ways to alleviate IUGR-induced damage to the small intestine of neonates is therefore critical.

Emerging evidence now indicates that polydatin (PD, also known as piceid) is a promising therapeutic treatment for intestinal injury [10–12]. This naturally occurring polyphenol has several health benefits, such as antioxidant and anti-inflammatory activities, and can be found in nonalcoholic grape juices and the traditional Chinese herb *Polygonum cuspidatum*. PD has potent bioactive properties, with antioxidant activities that can protect enterocytes against oxidative stress induced by hydrogen peroxide [11]. It may also serve as a protective substance to maintain mitochondrial function and facilitate the supply of energy in intestinal epithelial cells [11].

Recent studies have demonstrated that PD improves the features of intestinal damage in animal models [12–14]. In mice with acute colitis induced by dextran sulfate sodium, PD has been found to ameliorate the colitis symptoms and prevent oxidative stress and apoptosis in colon tissues [12]. In a mouse model of radiation-induced intestinal injury, PD effectively preserved mucosal morphology and inhibited the radiation-induced increase in intestinal endothelial apoptosis [13]. PD can also functionally regulate intestinal and systematic inflammatory responses [10, 14].

At present, however, little information is available regarding the use of PD as a nutritional supplement in IUGR offspring, and satisfactory strategies for the treatment of intestinal damage due to IUGR in neonates remain limited. Therefore, the present study was conducted using the piglet as a model to test the hypothesis that dietary supplementation with PD may have the potential to protect against small intestinal injury induced by IUGR. Notably, the spontaneous IUGR piglet is currently recognized as an ideal model for the research on IUGR in human infants, as increasing evidence now points to physiological and metabolic similarities between swine and humans [15]. In this regard, this study may help develop appropriate therapeutic approaches for treating intestinal disorders in the IUGR neonates.

## 2. Materials and Methods

### 2.1. Experimental Animals and Design

**Figure 1:**
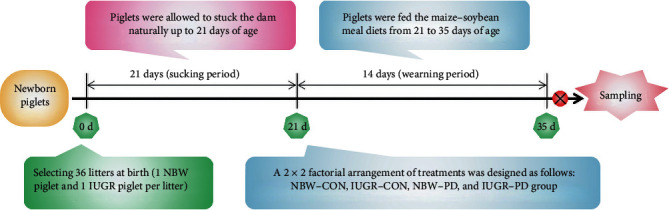
Schematic representation of the experimental procedures. IUGR: intrauterine growth retardation; IUGR-CON: intrauterine growth-retarded piglets fed a basal diet; IUGR-PD: intrauterine growth-retarded piglets fed a polydatin-supplemented diet; NBW: normal birth weight; NBW-CON: normal birth weight piglets fed a basal diet; NBW-PD: normal birth weight piglets fed a polydatin-supplemented diet.

The animal experimentation and the corresponding protocols performed in this study (Permit number SYXK-2017-0027) were approved by the Nanjing Agricultural University Institutional Animal Care and Use Committee. Crossbred piglets (Duroc × [Landrace × Yorkshire]) were obtained from sows of the experimental herd of the Anyou Biotechnology Group Co., Ltd. (Taicang, Jiangsu, China). Piglets with a BW near the mean value of the herd (less than 0.5 SD) were identified as the normal BW (NBW) piglets, and the IUGR piglets were defined as having a 2 SD lower BW [4, 16, 17]. At birth, 36 female NBW piglets (1.56 ± 0.08) and an equal number of same-sex IUGR littermates (0.91 ± 0.10) were selected from 36 litters (one NBW piglet and one IUGR piglet per litter) from the herd, for a total of 72 neonatal piglets. All piglets were weaned at 21 days of age and then moved from the farrowing room to the weaner unit. A 2 × 2 factorial design was used with two levels of BW factors (i.e., NBW and IUGR) and two levels of diet factors (i.e., fed either a basal diet [CON] or a diet supplemented with PD at a dosage of 250 mg/kg [PD]). Each treatment had six replicates (pens) and each replicate had 6 piglets (Figure 1). The composition and nutrient levels of the basal diet are listed in Supplementary Table 1. The body weight and feed intake of piglets in each pen were recorded at 21 and 35 days of age to calculate their average daily gain (ADG), average daily feed intake (ADFI), and feed efficiency (FE) during the entire period of feeding trial.

### 2.2. Sample Collection

At 35 days of age, one piglet was randomly selected from each replicate of per treatment for blood sample collection after overnight fasting. Heparinized blood was collected from the precaval vein of piglets and its plasma was obtained by centrifugation. The selected piglets were then euthanized by exsanguination after electrical stunning, and the small intestine was immediately collected. Two continuous sections (1.5 cm) were collected from the middle position of the whole jejunum. One sample was fixed in 4% paraformaldehyde (#G1101; Wuhan Servicebio Technology Co., Ltd., Wuhan, Hubei, China), and the other one was immersed in *in situ* hybridization fixing solution (#G1113; Wuhan Servicebio Technology Co., Ltd.). An additional jejunum section (approximately 20 cm) was cut from the adjacent position for the collection of mucosal samples. The plasma and mucosal samples were stored in −80°C freezers for subsequent biochemical analysis.

### 2.3. Plasma Parameters

Circulating diamine oxidase (DAO) activity was analyzed using a colorimetric assay kit (#A088-1-1; Nanjing Jiancheng Institute of Bioengineering, Nanjing, Jiangsu, China) based on the spectrophotometry method of Hosoda et al. [18]. The content of D-lactate in plasma was determined using a commercial kit (#AAT-13811) obtained from AAT Bioquest (Sunnyvale, CA, USA). All procedures were performed with strict adherence to the manufacturer's instructions.

### 2.4. Intestinal Morphology Observation

Jejunal segments were dehydrated and embedded in paraffin after fixation in paraformaldehyde solution for 24 h. Specimens were sliced into 5 *μ*m thick sections and stained with hematoxylin and eosin for morphological evaluation. Twenty well-oriented and intact villi and adjacent crypts per slide were randomly selected for the evaluation of jejunal mucosal morphology. Villus height (VH) and crypt depth (CD) were determined by microscopy (Nikon 80i; Nikon, Tokyo, Japan) by an assessor blinded to the treatments.

### 2.5. Fluorescence *In Situ* Hybridization (FISH)

Paraffin-embedded jejunal tissue slides (5 *μ*m) were deparaffinized and hybridized to the universal eubacterial oligonucleotide probe EUB-338 (5′-GCTGCCTCCCGTAGGAGT-3′) or to a control probe NONEUB-338 (5′-CGACGGAGGGCATCCTCA-3′), according to the method described previously [19]. The 5-prime ends of the probes were labeled with Cy3 (EUB-338) or FAM (NONEUB-338). The Universal *In Situ* Hybridization Detection Kit III (#MK1031) was used for FISH in this study. All procedures were performed in accordance with the experimental protocols provided by the manufacturer (Boster, Wuhan, China). Stained bacteria were observed with a fluorescence microscope (Nikon 80i).

### 2.6. Terminal Deoxynucleotidyl Transferase-Mediated dUTP Nick End Labeling (TUNEL)

The percentage of apoptotic cells was measured using a TUNEL assay kit (#A113-03), according to the manufacturer's guidelines (Vazyme, Nanjing, Jiangsu, China). Paraffin sections were deparaffinized and then pretreated with proteinase K (20 *μ*g/mL dissolved in phosphate-buffered saline) at room temperature for 20 min. The slides were then incubated with the TUNEL reagents in the dark at 37°C for 1 h. The nuclei were identified by staining with 4′-6-diamidino-2-phenylindole (DAPI) dissolved in antifade mounting medium (#P0131; Beyotime, Shanghai, China). The number of TUNEL-labeled cells in fifteen randomly fields per section was quantified by a blinded observer using a fluorescence microscope.

### 2.7. Mucosal Redox and Immune Status

Approximately 300 mg of frozen jejunal mucosa samples was placed in 1 : 9 (wt/vol) saline solution and then homogenized with a high-speed benchtop homogenizer (Tekmar, Cincinnati, OH, USA). The resulting mucosal homogenate was centrifuged at 15,000 *g* for 10 min at 4°C, and the supernatant was used for the analysis of superoxide dismutase (SOD; #A001-1-2), glutathione peroxidase (GSH-Px; #A005-1-2), catalase (CAT; #A007-1-1), glutathione S-transferase (GST; #A004-1-1), glutathione reductase (GR; #A062-1-1), and myeloperoxidase (MPO; #A044-1-1) activities and reduced glutathione (GSH; #A006-1-1) and malondialdehyde (MDA; #A003-1-2) concentrations with colorimetric kits purchased from Nanjing Jiancheng Institute of Bioengineering. For the detection of 8-hydroxy-2′-deoxyguanosine (8-OHDG), total DNA of jejunal mucosa was extracted with a Genomic DNA Isolation Kit (#K281-50; BioVision, Milpitas, CA, USA) and the 8-OHDG content in the DNA samples was tested using a 8-OHDG Enzyme-Linked Immunosorbent Assay (ELISA) Detection Kit (#589320-96S) following the manufacturer's protocol (Cayman Chemical, Ann Arbor, MI, USA). The concentrations of tumor necrosis factor alpha (TNF-*α*; CSB-E16980p) and interleukin-10 (IL-10; CSB-E06779p) were determined by porcine-specific ELISA kits obtained from CUSABIO Biotech (Wuhan, China). The detection ranges were 0.047 to 3 ng/mL for TNF-*α* and 6.25 to 400 pg/mL for IL-10, respectively. The minimum detectable doses were less than 0.040 ng/mL for TNF-*α* and 1.56 pg/mL for IL-10. The inter- and intra-assay coefficients of variance of the assay kits were less than 8% and 10%, respectively.

Protein concentrations of the samples were determined according to the manufacturer's instructions using an Enhanced Bicinchoninic Acid (BCA) Protein Assay Kit (#P0010; Beyotime). The data of each sample were normalized using the protein content to allow intersample comparisons.

### 2.8. Transmission Electron Microscopy

Jejunum samples were fixed in 2.5% glutaraldehyde in 0.1 M sodium cacodylate buffer overnight at 4°C and then postfixed with 1% osmium tetroxide in 0.2 M phosphate-buffered saline for 2 h at 4°C. Following which, the sections were dehydrated with a graded series of ethanol solutions, rinsed in propylene oxide, and embedded in epoxy resin. Thin sections were cut with a Reichert Ultracut microtome (Leica, Deerfield, IL, USA) and observed and photographed using a transmission electron microscope (Hitachi H-7650, Tokyo, Japan).

### 2.9. Detection of Mitochondrial DNA (mtDNA) Content

After the isolation of total DNA as described above, the copy number of mtDNA in each DNA sample was measured by coamplifying the mitochondrial D-loop (mtD-loop) and beta actin (ACTB) genes by quantitative PCR using the QuantStudio 5 Real-Time PCR System and the SYBR Green reagent (#Q311-02; Vazyme). The following primer sequences were used: mtD-loop sense primer GATCGTACATAGCACATATCATGTC and anti-sense primer GGTCCTGAAGTAAGAACCAGATG and ACTB sense primer CCCCTCCTCTCTTGCCTCTC and anti-sense primer AAAAGTCCTAGGAAAATGGCAGAAG. The 2^-*ΔΔ*Ct^ method was performed to calculate the copy number of mtDNA [20].

### 2.10. Measurement of Mitochondrial Enzyme Activities

Mitochondria were isolated from jejunum mucosa with the Tissue Mitochondria Isolation Kit (#C3606; Beyotime). After quantifying the protein content of the mitochondrial suspension, the activities of citrate synthase (CS; #BC1065), electron transport chain (ETC) complexes I (#BC0515), II (#BC3230), III (#BC3240), and IV (#BC0945), and adenosine triphosphate (ATP) synthase (#BC1445) were determined by colorimetric kits following the guidelines of the manufacturer (Solarbio, Beijing, China).

### 2.11. Determination of Mucosal ATP Content

Jejunal mucosa samples were weighed, ground, blended with boiling double-distilled H_2_O, and boiled for 30 min. After centrifugation, the supernatant was collected to determine the ATP content of each sample with a commercial kit (#A095-1-1; Nanjing Jiancheng Institute of Bioengineering). The results were expressed as *μ*mol/g wet weight.

### 2.12. Sirtuin 1 (SIRT1) Activity

Jejunal nuclear fractions were prepared based on a method described by Park et al. [21]. The deacetylase activity of SIRT1 in the jejunal mucosa was measured with a SIRT1 Activity Detection Kit (#50287.2) according to the recommended protocols of the manufacturer (Genmed, Shanghai, China).

### 2.13. Gene Expression

Total RNA was extracted from jejunal mucosa samples using the Total RNA Isolation Reagent (#R401-01; Vazyme). Determination of the yields, quality, and integrity of isolated RNA samples were performed based on the method reported by Li et al. [8]. After that, the first-strand complementary DNA (cDNA) was synthesized from total RNA with the High-Capacity cDNA Reverse Transcription Kit (#RR036A; Takara Biotechnology, Dalian, Liaoning, China). Real-time PCR reactions were performed following the recommended process of the manufacturer (Vazyme).  Table 1 includes the details of the primer sequences used for the following target and reference genes: occludin (*OCLN*), zonula occludens 1 (*ZO1*), claudin 1 (*CLDN1*), claudin 2 (*CLDN2*), claudin 3 (*CLDN3*), glyceraldehyde-3-phosphate dehydrogenase, and *ACTB*. The fold changes in the expression of specific mRNAs were calculated using the 2^-*ΔΔ*Ct^ method [20].

### 2.14. Western Blotting

Jejunal mucosa samples were lysed in ice-cold radioimmunoprecipitation assay reagent (#P0013B; Beyotime) containing protease inhibitors (#ST506; Beyotime) and phosphatase inhibitors (#P2850; Sigma-Aldrich, St. Louis, MO, USA). Protein solutions were obtained by centrifugation and then mixed with 5× sodium dodecyl sulfate sample buffer (#P0015; Beyotime). The mixture was boiled for 5 min. After determining protein content with the BCA detection kit, an equal amount of extracted protein was separated by polyacrylamide gel electrophoresis in the presence of sodium dodecyl sulfate. The separated proteins were then transferred to an Immobilon-P transfer membrane (#IPVH00010; Millipore, Bedford, MA, USA). The membranes were blocked with 5% skimmed dry milk in Tris-buffered saline combined with 0.2% Tween-20 (TBST) at room temperature for 2 h. After three washes in TBST, the blots were incubated with the following primary antibodies at room temperature for 4 h: antiphosphorylated adenosine monophosphate-activated protein kinase alpha (AMPK*α*, phosphorylated at Thr^172^) (#2535S; CST, Danvers, MA, USA; 1 : 1,000 dilution), anti-AMPK*α* (#2532S; CST; 1 : 1,000 dilution), anti-SIRT1 (#NBP2-27205; Novus Biologicals, Littleton, CO, USA; 1 : 750 dilution), antiperoxisome proliferation-activated receptor gamma coactivator 1 alpha (PGC-1*α*; #ab106814; Abcam, Cambridge, MA, USA; 1 : 1,000 dilution), and ACTB (#4970S; CST; 1 : 1,000 dilution). After incubation with appropriate secondary antibodies, the immunoreactive proteins were detected with an enhanced chemiluminescence HRP substrate (#WBKLS0100; Millipore, Bedford, MA, USA). The gray scale values of bands were measured using the Gel-Pro Analyzer program (version 4.0, Media Cybernetics, Inc., MD, USA).

### 2.15. Statistical Analysis

The Statistical Package for the Social Sciences (SPSS Inc., version 22.0; IBM Corporation, Armonk, NY, USA) was used for statistical analysis. The data were subjected to a two-way analysis of variance using the general linear model procedure to analyze the main effects of BW and diet and their interaction (*B* × *D*). A *P* value of less than 0.05 was considered statistically significant. Duncan's post hoc test was used to explore all significant differences. All data are expressed as the mean and their pooled standard error (SEM).

## 3. Results

### 3.1. Growth Performance

Relative to the NBW piglets, IUGR decreased the initial (*P* < 0.001) and final body weights (*P* < 0.001) of piglets and reduced their ADG (*P* < 0.001) and ADFI (*P* = 0.001) during the first two weeks after weaning (Table 2). Supplementation with PD had no effect on body weights, ADG, ADFI, or FE of weanling piglets regardless of BW (*P* > 0.05). A significant interaction of BW and diet was observed for the ADG of piglets; the decrease in ADG in the IUGR piglets was not observed when IUGR piglets were supplemented with PD (*P* = 0.035).

### 3.2. Intestinal Barrier Function

**Figure 2:**
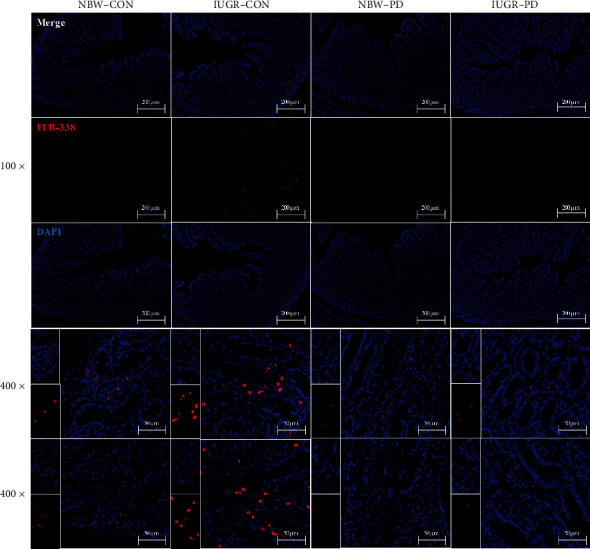
Representative micrographs of bacteria colonization detected by FISH using the Cy3-labeled oligonucleotide EUB-338. DAPI: 4′-6-diamidino-2-phenylindole; FISH: fluorescence *in situ* hybridization; IUGR-CON: intrauterine growth-retarded piglets fed a basal diet; IUGR-PD: intrauterine growth-retarded piglets fed a polydatin-supplemented diet; NBW-CON: normal birth weight piglets fed a basal diet; NBW-PD: normal birth weight piglets fed a polydatin-supplemented diet.

The FISH analysis using the Cy3-labeled oligonucleotide EUB-338 revealed an increased bacterial colonization in the jejunal crypts of IUGR-CON piglets (Figure 2). Conversely, the jejunal sections from the IUGR-PD group showed decreased fluorescence signals for eubacterial staining to levels similar to those of their NBW counterparts. Monitoring of intestinal integrity by D-lactate concentration and DAO activity in the plasma revealed higher DAO activity (*P* = 0.001) and D-lactate concentration (*P* < 0.001) in the IUGR piglets than in their NBW littermates (Table 3). The PD-supplemented diet significantly reduced the content of circulating D-lactate (*P* = 0.039) when compared with the basal diet. In addition, the increments in DAO activity (*P* = 0.039) and D-lactate concentration (*P* = 0.004) in the IUGR piglets were reversed by supplementation with PD.

### 3.3. Jejunal Tight Junction Expression

The mRNA abundance of *OCLN* (*P* = 0.001) was significantly reduced by IUGR in the piglet jejunum (Table 4). By contrast, supplementation with PD increased the expression of jejunal *OCLN* (*P* = 0.004). PD supplementation also reversed the IUGR-induced increase in the mRNA level of *CLDN2* (*P* = 0.013). Neither BW nor diet affected the mRNA expression of jejunal *ZO1*, *CLDN1*, or *CLDN3* in the piglet jejunum (*P* > 0.05).

### 3.4. Jejunal Morphology

Relative to the NBW piglets, the IUGR piglets showed a decrease in jejunal VH (*P* = 0.001; Table 5). PD increased jejunal VH (*P* = 0.033) and the VH : CD ratio (*P* = 0.049) of piglets relative to those fed a basal diet. No difference was found in the jejunal CD of piglets among the groups (*P* > 0.05).

### 3.5. Jejunal Apoptotic Rate and Caspase Activities

**Figure 3:**
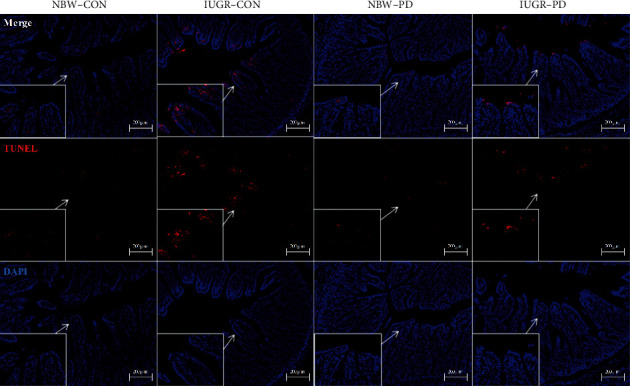
Representative micrographs of TUNEL staining carried out on paraformaldehyde-fixed jejunal sections of weanling piglets. DAPI: 4′-6-diamidino-2-phenylindole; IUGR-CON: intrauterine growth-retarded piglets fed a basal diet; IUGR-PD: intrauterine growth-retarded piglets fed a polydatin-supplemented diet; NBW-CON: normal birth weight piglets fed a basal diet; NBW-PD: normal birth weight piglets fed a polydatin-supplemented diet; TUNEL: terminal deoxynucleotidyl transferase-mediated dUTP nick end labeling.

An increased apoptotic rate (*P* = 0.001; Figure 3) was noted in the jejunum of IUGR piglets relative to their NBW counterparts (Table 6), along with increased caspase-3 (*P* = 0.010) and caspase-9 (*P* = 0.011) activities. Supplementation with PD reduced the percentage of jejunal apoptosis (*P* = 0.024) and the activity of caspase-9 (*P* = 0.009) in comparison with the basal diet. The IUGR piglets showed decreased apoptotic rate (*P* = 0.008) and caspase-3 activity (*P* = 0.019) when supplemented with PD.

### 3.6. Jejunal Redox Status

IUGR induced a decrease in the GSH content (*P* = 0.001) and increases in MDA (*P* = 0.023) and 8-OHDG formation (*P* = 0.017) in the jejunum of IUGR piglets when compared with their NBW counterparts (Table 7). Dietary PD supplementation increased the activities of jejunal SOD (*P* = 0.026) and GST (*P* = 0.006) and inhibited the generation of MDA (*P* = 0.015) and 8-OHDG (*P* = 0.034). Two-way ANOVA showed an obvious interaction between BW and diet for the concentration of MDA (*P* = 0.006). IUGR significantly elevated the MDA concentration in piglets fed a basal diet, but this increase was suppressed by PD supplementation.

### 3.7. Jejunal Inflammatory Responses

The activity of jejunal MPO (*P* = 0.010) and the TNF-*α* content (*P* = 0.003) were substantially increased in the IUGR group compared with the NBW group (Table 8). Supplementation with PD decreased TNF-*α* concentration (*P* = 0.003) and inhibited the IUGR-induced increase in MPO activity (*P* = 0.029) in the jejunum. No difference was observed in the amount of jejunal IL-10 protein among the groups (*P* > 0.05).

### 3.8. Jejunal Energy Metabolism

When compared with NBW piglets, IUGR piglets showed inhibited CS (*P* = 0.021) and complex I (*P* = 0.029) activities in the jejunum, along with a reduction in ATP production (*P* = 0.017; Table 9). Dietary PD supplementation increased the CS activity (*P* = 0.020) and improved the ATP generation (*P* = 0.009) in the jejunum irrespective of BW. PD supplementation increased the activities of complex III (*P* = 0.039) and ATP synthase (*P* = 0.006) in the IUGR jejunum. Neither BW nor diet affected the activity of complex II or IV among the groups (*P* > 0.05).

### 3.9. Jejunal Mitochondrial Structure

**Figure 4:**
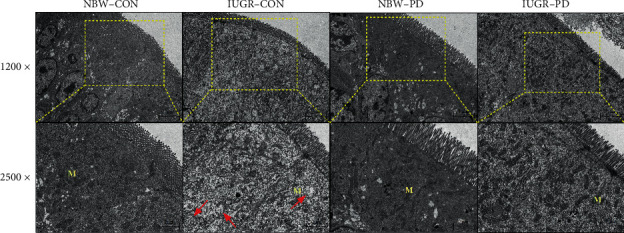
Effect of dietary polydatin supplementation on the jejunal mitochondrial ultrastructure of weanling piglets with different birth weights. Red arrows indicated the swelling of mitochondria. IUGR-CON: intrauterine growth-retarded piglets fed a basal diet; IUGR-PD: intrauterine growth-retarded piglets fed a polydatin-supplemented diet; M: mitochondrion; NBW-CON: normal birth weight piglets fed a basal diet; NBW-PD: normal birth weight piglets fed a polydatin-supplemented diet.

The mitochondria of enterocytes in the NBW piglets were intact with dense matrix and regular cristae (Figure 4). By contrast, in the IUGR-CON piglets, quite a few mitochondria were markedly damaged with abnormal shape and disorganized cristae, probably due to oedema in the matrix of mitochondria. After a two-week administration of PD, the severity of mitochondrial swelling in enterocytes was effectively improved.

### 3.10. Jejunal Mitochondrial Biogenesis and the Associated Signaling Pathway

**Figure 5:**
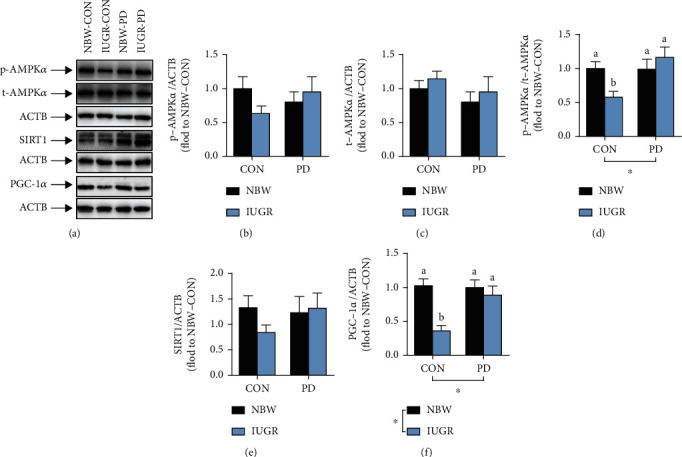
Effect of dietary polydatin supplementation on the protein expression of jejunal total AMPK*α* (b), phosphorylated AMPK*α* (c), SIRT1 (e), and PGC-1*α* (f) and the relative phosphorylation of AMPK*α* (d) in weanling piglets with different birth weights. Data are presented as the means and SEM, *n* = 6. ^∗^*P* < 0.05. Different letters above the bar indicate a significant difference (*P* < 0.05). ACTB: beta actin; CON: basal diet; IUGR: intrauterine growth retardation; mtDNA: mitochondrial DNA; NBW: normal birth weight; p-AMPK*α*: phosphorylated adenosine monophosphate-activated protein kinase alpha; PD: polydatin-supplemented diet; PGC-1*α*: peroxisome proliferation-activated receptor gamma coactivator 1 alpha; SIRT1: sirtuin 1; t-AMPK*α*: total adenosine monophosphate-activated protein kinase alpha.

Piglets in the IUGR group exhibited significantly decreased mtDNA copy number (*P* = 0.003; Table 10) and PGC-1*α* protein expression (*P* = 0.004; Figure 5) in the jejunum compared with their NBW littermates. However, IUGR had no effect on SIRT1 activity, the expression levels of phosphorylated AMPK*α* (p-AMPK*α*), total AMPK*α* (t-AMPK*α*), and SIRT1, or the ratio of p-AMPK*α* to t-AMPK*α* (*P* > 0.05). PD supplementation promoted SIRT1 activity (*P* = 0.028), increased the relative phosphorylation level of AMPK*α* (*P* = 0.034), and upregulated the protein amount of PGC-1*α* (*P* = 0.032) in comparison with the basal diet. PD supplementation also reversed the IUGR-induced reductions in jejunal mtDNA copy number (*P* = 0.048) and the protein contents of phosphorylated AMPK*α* (*P* = 0.027) and PGC-1*α* (*P* = 0.033).

## 4. Discussion

The present findings demonstrated that IUGR was detrimental to the jejunal barrier function of weanling piglets, as evidenced by a greater intestinal permeability, increased bacterial translocation, and disturbed expression patterns of tight junction genes. Disruption of the jejunal barrier function increases the exposure risk of young piglets to exogenous pathogens, antigens, and other noxious factors, which may in turn promote mucosal injury and systemic inflammation [7]. This impaired barrier function may account for the increases in jejunal 8-OHDG and MDA contents, MPO activity, and TNF-*α* secretion observed in the IUGR piglets. These changes in the jejunum of the IUGR piglets were signs of oxidative stress and inflammation that can promote the occurrence and development of small intestinal injury [22].

The decreased mtDNA content and the lower efficiency of ATP production noted in the jejunum of IUGR piglets could also possibly reflect a disturbance in mitochondrial biogenesis and oxidative metabolism. Mitochondrial dysfunction limits the availability of ATP for both nutrient absorption and the renewal of the intestinal epithelium, resulting in malabsorption and intestinal disorders [23]. Collectively, these effects of IUGR act in concert to incite the small intestinal injury observed in the IUGR piglets and may therefore be part of potential mechanisms that explain the inferior growth performance of the IUGR piglets during the early period after weaning.

After the two-week feeding trial, PD supplementation was found to increase the ADG and FE of piglets, which may be associated with the beneficial roles of PD in restoring jejunal mucosal damage and barrier dysfunction. In the current study, PD supplementation showed potential to inhibit the excessive apoptosis of villus cells due to IUGR. Similarly, rodent studies have indicated that PD is effective in alleviating different harmful stimuli-induced apoptosis in the small intestine [14], the liver [24], and the heart [25]. Under stressful circumstances, PD can inhibit the overactivation of the apoptotic signaling pathway by stabilizing caspase cascades and the expression of proapoptotic and antiapoptotic regulators [26, 27]. Consistently, in the present study, supplementation of IUGR piglets with PD resulted in lower activity of jejunal caspase-9, which is an initiator caspase in the mitochondrial-dependent apoptotic pathway [28]. These results implied that PD may alleviate the IUGR-induced enterocyte apoptosis, probably through its protective action that blocks the overactivation of apoptosis signals associated with mitochondrial dysfunction. Our findings therefore confirm the previous findings reported on a rodent model regarding small intestinal damage, where PD was proposed to protect mitochondria and to counteract the higher apoptosis of enterocytes due to hemorrhagic shock [11].

Excessive apoptosis of the epithelial cells not only induces mucosal villus atrophy but it also causes the formation of “bare areas” in the epithelial layer that would provide attachments and penetration sites for bacteria on the lamina propria [29]. In the present study, a decrease in apoptosis of epithelial cells in IUGR-PD piglets appeared to alleviate the jejunal barrier dysfunction and mucosal damage, as indicated by the lower DAO activity and D-lactate concentration in the plasma. DAO is an intracellular enzyme that is particularly abundant in the villi of the small intestine and it is released into the circulation when mucosal epithelial cells and intestinal integrity are impaired [30]. Similarly, D-lactate is a byproduct of bacterial metabolism and its accumulation in the blood circulation can reflect the severity of intestinal barrier dysfunction [31]. Furthermore, the beneficial effect of PD on the mucosal barrier function was also confirmed by FISH analysis, which showed a reduced expression of bacterial rRNA in the jejunal crypts of the IUGR piglets supplemented with PD.

Treatment with PD increased the expression of *OCLN* in the jejunum of piglets, which may provide another line of evidence for the maintenance of jejunal barrier function. OCLN is an important extracellular component of the tight junction and serves to restrict permeability to low-molecular-mass molecules while increasing the electrical resistance of epithelial barrier [32]. Ruan et al. [33] have shown that PD also protects the blood-brain barrier after stroke by upregulating the expression of ZO1 and OCLN. Moreover, PD treatment inhibited the upregulation of jejunal *CLDN2* in the IUGR piglets. CLDNs are mainly responsible for controlling paracellular transport through the distinct charge and size selectivity. Some members act by plugging the paracellular pathway, whereas others function as paracellular channels [34]. Among them, CLDN2 increases the permeability of epithelial barrier by promoting the expression of cation-selective pores, and its upregulation is frequently observed in the leaky epithelium [35]. Therefore, in addition to alleviating enterocyte apoptosis, the role of PD in regulating the *OCLN* and *CLDN2* expression may also promote the recovery of jejunal barrier function in the IUGR piglets.

In the present study, supplementation with PD reversed the increased activity of jejunal MPO observed in the IUGR piglets, suggesting that PD may alleviate the inflammation caused by the MPO system of neutrophils. Bacteria that enter the intestinal mucosa can trigger mucosal inflammation by stimulating neutrophil infiltration and MPO reactions. The MPO system of neutrophils plays a crucial role in host defense against bacterial pathogens by generating hypochlorous acid [36]. However, hypochlorous acid is a highly destructive and nonselective oxidant that reacts avidly with cellular macromolecules such as DNA and lipids. Consequently, prolonged MPO activation is implicated in the development of inflammation and oxidative stress in normal tissue and can lead to an eventual injury outcome in the small intestine [37].

In addition, PD may act as a potent anti-inflammatory agent by inhibiting the overproduction of jejunal TNF-*α*. Xu et al. [27] have shown that PD suppresses the secretion of proinflammatory cytokines, including TNF-*α*, IL-1*β*, and IL-6, into the circulation. The available literature has indicated that the anti-inflammatory effects of PD may be attributed to its ability to prevent the overactivation of nuclear factor-kappa B (NF-*κ*B) signals, as this signaling pathway is responsible for regulating the transcription of proinflammatory mediators during immunological stress [10, 38, 39]. Support for an inhibitory effect of PD on NF-*κ*B signals has been provided by Wu et al. [24], who observed that PD decreased the DNA binding ability of NF-*κ*B p65 during intestinal inflammation. Similarly, Ye et al. [38] demonstrated that PD inhibited the translocation of p65 protein from the cytoplasm to the nucleus, therefore preventing the overactivation of NF-*κ*B signals.

The antioxidant potency of PD is another key factor that promotes resistance to oxidative stress and the recovery of small intestinal damage. PD acts as a scavenger for free radicals as it contains hydroxyl groups. Among which, the 4′-hydroxyl group is the preferred reaction site as a result of the resonance effects that occur between the two aromatic rings [40–42]. Therefore, PD can quench a variety of free radicals produced both *in vitro* and *in vivo*, including 2,2-diphenyl-1-picryl-hydrazyl, 2,2-azobis (2-amidino-propane) dihydrochloride, superoxide anion, hydrogen peroxide, and hydroxyl radicals [27, 43, 44]. Notably, Fabris et al. [45] showed that PD is a suitable compound for the prevention and control of the lipid peroxidation because of its lipophilicity. These antioxidant effects of PD may decrease the risk of development of oxidative stress in the intestine and could parallel the decline in MDA and 8-OHDG levels observed in the jejunum of IUGR piglets supplemented with PD.

The present study also demonstrated that PT had the ability to facilitate enzymatic antioxidant mechanism with evidence of the increases in SOD and GST activities in the jejunum of piglets supplemented with PD. SOD serves as the first-line player in the antioxidant defense system and is responsible for eliminating superoxide anion, the precursor of other free radicals with high reactivity. Consistent with our findings, previous studies have also reported that PD can stimulate cellular antioxidant defenses by activating SOD, GSH-Px, GST, and CAT [46–48]. Moreover, the IUGR-induced reduction in jejunal GST activity was largely recovered by supplementation with PD. GST is an important cellular enzyme that can detoxify both exogenous and endogenous toxins by mediating the reaction between electrophiles and GSH [49]. Recently, PD has been demonstrated to function as an activator of nuclear factor erythroid 2-related factor 2 (NRF2), the master regulator that can activate the transcription of intracellular antioxidants and phase II detoxifying enzymes to control the cellular redox balance [50]. This finding may offer a further explanation for the ability of PD to stimulate SOD and GST activities, as both these enzymes are downstream of NRF2 signals [51]. Collectively, the anti-inflammatory and antioxidant properties of PD may assist in the mitigation of small intestinal inflammation and oxidative damage in the IUGR piglets.

Apart from its anti-inflammatory and antioxidant activities, PD may also alleviate IUGR-induced jejunal damage by a mechanism that promotes the efficiency of mitochondrial oxidative metabolism. The mitochondria are the major source of ATP in most eukaryotic cells, so they are vital organelles for energy homeostasis and cell survival. In the intestine, enterocytes have a high energy requirement as their roles are rapid renewal of epithelium and the active transport of nutrients. In this study, dietary PD supplementation was effective in increasing ATP generation and CS activity in the jejunum. CS is a critical enzyme that functions as the entry gate for acetyl CoA into the tricarboxylic acid cycle, thereby determining subsequent ATP formation [52]. The IUGR piglets showed decreased ETC complex III and ATP synthase activities, but dietary PD supplementation significantly restored their activities, suggesting the promotion of a higher flux through the ETC and an increase in intracellular ATP levels as a result.

Previous studies have consistently reported that PD can reverse the broad myocardial infarction-induced decreases in the activities of mitochondrial oxidative metabolic enzymes, such as CS, and in the functioning of the ETC complexes [25]. A recent study of *Musca domestica* larvae confirmed that PD could also restore mitochondrial membrane potential, increase oxidative phosphorylation efficiency, and facilitate ATP production in response to cadmium-induced heavy metal stress [53]. In the present study, the numbers of swollen mitochondria were substantially reduced by PD supplementation, further confirming previous findings that PD ameliorates mitochondrial ultrastructural injury *in vitro* [25, 54, 55]. Therefore, the beneficial responses may aid in the recovery of the intestinal epithelium from stress-associated damage in the IUGR neonates.

The induction of AMPK*α* phosphorylation after PD treatment may contribute to the improved mitochondrial function and ATP supply in the small intestine of IUGR piglets. AMPK functions as a regulator of the metabolic adaptations in response to energy deprivation by acting in coordination with another metabolic sensor SIRT1, a nicotinamide adenine dinucleotide- (NAD^+^-) dependent deacetylase [56]. The activation of AMPK stimulates the activity of SIRT1 by enhancing the cellular NAD^+^ concentration [56]. This effect may explain the increase in jejunal SIRT1 activity observed in the piglets after supplementation with PD.

Similarly, in a rat model of acute hemorrhagic shock, PD has been demonstrated to prevent the decreases in SIRT1 expression and activity [55]. SIRT1 can improve mitochondrial function by activating several transcriptional regulators, including PGC-1*α*, which synergistically facilitates mitochondrial biogenesis and respiration rates and increases substrate availability for ATP generation [56]. In this regard, we infer that the AMPK/SIRT1 signaling pathway and its downstream PGC-1*α* may be part of the mechanism by which PD activates mitochondrial CS, complex III, and ATP synthase to increase ATP generation in the jejunum of IUGR piglets. In agreement with this idea, Chen and Lan [57] have shown that PD is capable of promoting AMPK phosphorylation and increasing SIRT1 expression.

PD also has the potential to improve glucose and lipid metabolisms in insulin-resistant HepG2 cells by activating AMPK signals [58]. Zeng et al. [14] have demonstrated that PD is a potent SIRT1 activator and stimulates the SIRT1/PGC-1*α*/SOD2 axis to alleviate small intestine injury following severe hemorrhagic shock. Thus, the benefits of PD in mitigating jejunal damage may be mediated at least in part by the combined actions of AMPK, SIRT1, and PGC-1*α*, which form an orchestrated network to maintain mitochondrial function and cellular energy homeostasis in the small intestine.

## 5. Conclusions

The data obtained from the present study indicates that PD supplementation confers protection against IUGR-induced jejunal injury and barrier dysfunction, probably by mitigating oxidative damage and inflammatory responses, restoring the expression of tight junction complexes, and facilitating mitochondrial biogenesis and oxidative metabolism. This study provides important support for using PD as a nutritional supplement to alleviate the small intestinal damage observed in IUGR neonates.

## Figures and Tables

**Table 1:** Primer sequences used for real-time PCR assay.

Name^1^	GenBank^2^	Sequence^3^ (5′ → 3′)	Length (bp)
*ZO1*	XM_021098827.1	AGGCGTGTTTAACAGCAACG	182
		CCAAAGGACTCAGCAGGGTT	
*OCLN*	NM_001163647.2	CAGGTGCACCCTCCAGATTG	167
		ATGTCGTTGCTGGGTGCATA	
*CLDN1*	NM_001244539.1	ACAGGAGGGAAGCCATTTTCA	82
		TTTAAGGACCGCCCTCTCCC	
*CLDN2*	NM_001161638.1	GGATCCTGCGGGACTTCTAC	153
		TGGAGCGATTTCCTTGCAGT	
*CLDN3*	NM_001160075.1	GAGACCAGTCCACCCAGATG	147
		AGGTTTCATGGTCCGTGCTG	
*GAPDH*	NM_001206359.1	CCAAGGAGTAAGAGCCCCTG	125
		AAGTCAGGAGATGCTCGGTG	
*ACTB*	XM_003124280.5	TGGAACGGTGAAGGTGACAG	176
		CTTTTGGGAAGGCAGGGACT	

^1^ *ACTB*: beta actin; *CLDN1*: claudin 1; *CLDN2*: claudin 2; *CLDN3*: claudin 3; *GAPDH*: glyceraldehyde-3-phosphate dehydrogenase; *OCLN*: occludin; *ZO1*: zonula occludens 1. ^2^GenBank accession number. ^3^Shown as the forward primer followed by the reverse primer.

**Table 2:** Effect of dietary polydatin supplementation on the growth performance of weanling piglets with different birth weights from 21 to 35 days of age.

Items^1,2^	CON		PD		SEM	*P* value		
	NBW	IUGR	NBW	IUGR		BW	Diet	*B* × *D*
Initial body weight (kg)	6.64^a^	4.83^b^	6.66^a^	4.81^b^	0.20	<0.001	0.983	0.916
Final body weight (kg)	9.45^a^	6.69^b^	9.39^a^	7.19^b^	0.28	<0.001	0.300	0.194
ADG (g/d)	200.20^a^	132.94^c^	195.24^ab^	169.84^b^	7.04	<0.001	0.101	0.035
ADFI (g/d)	286.11^a^	209.92^b^	280.36^a^	251.98^a^	8.66	0.001	0.171	0.076
FE (g/g)	0.70	0.63	0.70	0.68	0.01	0.120	0.373	0.465

^1^Data are presented as the means and SEM, *n* = 6. Mean values with different letters indicate a significant difference (*P* < 0.05). ^2^ADG: average daily gain; ADFI: average daily feed intake; BW: birth weight; *B* × *D*: the interaction effect of the main effects (BW and diet); CON: basal diet; FE: feed efficiency; IUGR: intrauterine growth retardation; NBW: normal birth weight; PD: polydatin-supplemented diet.

**Table 3:** Effect of dietary polydatin supplementation on the plasma diamine oxidase activity and D-lactate content of weanling piglets with different birth weights.

Items^1,2^	CON		PD		SEM	*P* value		
	NBW	IUGR	NBW	IUGR		BW	Diet	*B* × *D*
DAO (U/mL)	26.95^b^	102.84^a^	36.67^b^	58.55^b^	8.35	0.001	0.174	0.039
D-Lactate (nmol/mL)	435.59^b^	891.20^a^	496.69^b^	564.18^b^	46.18	<0.001	0.039	0.004

^1^Data are presented as the means and SEM, *n* = 6. Mean values with different letters indicate a significant difference (*P* < 0.05). ^2^BW: birth weight; *B* × *D*: the interaction effect of the main effects (BW and diet); CON: basal diet; DAO: diamine oxidase; IUGR: intrauterine growth retardation; NBW: normal birth weight; PD: polydatin-supplemented diet.

**Table 4:** Effect of dietary polydatin supplementation on the mRNA levels of jejunal tight junction complexes in weanling piglets with different birth weights.

Items^1,2^	CON		PD		SEM	*P* value		
	NBW	IUGR	NBW	IUGR		BW	Diet	*B* × *D*
*ZO1*	1.00^ab^	0.68^b^	1.03^a^	0.96^ab^	0.06	0.090	0.171	0.279
*OCLN*	1.00^b^	0.54^c^	1.53^a^	0.91^bc^	0.10	0.001	0.004	0.579
*CLDN1*	1.00	1.13	1.04	0.59	0.11	0.456	0.259	0.187
*CLDN2*	1.00^b^	1.60^a^	1.26^ab^	0.91^b^	0.10	0.489	0.233	0.013
*CLDN3*	1.00	1.09	1.30	1.11	0.11	0.839	0.486	0.550

^1^Data are presented as the means and SEM, *n* = 6. Mean values with different letters indicate a significant difference (*P* < 0.05). ^2^BW: birth weight; *B* × *D*: the interaction effect of the main effects (BW and diet); *CLDN1*: claudin 1; *CLDN2*: claudin 2; *CLDN3*: claudin 3; CON: basal diet; IUGR: intrauterine growth retardation; NBW: normal birth weight; *OCLN*: occludin; PD: polydatin-supplemented diet; *ZO1*: zonula occludens 1.

**Table 5:** Effect of dietary polydatin supplementation on the jejunal morphology of weanling piglets with different birth weights.

Items^1,2^	CON		PD		SEM	*P* value		
	NBW	IUGR	NBW	IUGR		BW	Diet	*B* × *D*
VH (*μ*m)	451.63^a^	359.04^b^	479.05^a^	422.55^a^	13.14	0.001	0.033	0.376
CD (*μ*m)	198.18	174.88	180.87	183.08	5.81	0.384	0.704	0.294
VH : CD (*μ*m/*μ*m)	2.31^ab^	2.08^b^	2.70^a^	2.34^ab^	0.09	0.071	0.049	0.677

^1^Data are presented as the means and SEM, *n* = 6. ^2^BW: birth weight; *B* × *D*: the interaction effect of the main effects (BW and diet); CD: crypt depth; CON: basal diet; IUGR: intrauterine growth retardation; NBW: normal birth weight; PD: polydatin-supplemented diet; VH: villus height; VH : CD: the ratio of villus height to crypt depth.

**Table 6:** Effect of dietary polydatin supplementation on the jejunal apoptosis and caspase activities of weanling piglets with different birth weights.

Items^1,2^	CON		PD		SEM	*P* value		
	NBW	IUGR	NBW	IUGR		BW	Diet	*B* × *D*
Apoptosis (%)	2.68^b^	8.20^a^	3.07^b^	3.98^b^	0.59	0.001	0.024	0.008
Caspase-3 (U/mg protein)	22.12^b^	43.48^a^	24.74^b^	25.92^b^	2.55	0.010	0.073	0.019
Caspase-8 (U/mg protein)	6.79	10.71	8.35	7.29	0.67	0.269	0.470	0.062
Caspase-9 (U/mg protein)	14.15^ab^	18.79^a^	9.59^b^	13.99^ab^	1.01	0.011	0.009	0.940

^1^Data are presented as the means and SEM, *n* = 6. Mean values with different letters indicate a significant difference (*P* < 0.05). ^2^BW: birth weight; *B* × *D*: the interaction effect of the main effects (BW and diet); CON: basal diet; IUGR: intrauterine growth retardation; NBW: normal birth weight; PD: polydatin-supplemented diet.

**Table 7:** Effect of dietary polydatin supplementation on the jejunal redox status of weanling piglets with different birth weights.

Items^1,2^	CON		PD		SEM	*P* value		
	NBW	IUGR	NBW	IUGR		BW	Diet	*B* × *D*
SOD (U/mg protein)	62.85^ab^	52.99^b^	70.58^a^	68.13^a^	2.62	0.210	0.026	0.445
GSH-Px (U/mg protein)	60.97	57.82	65.95	51.20	3.18	0.177	0.899	0.376
CAT (U/mg protein)	7.77	8.14	8.39	6.87	0.26	0.267	0.526	0.075
GST (U/mg protein)	79.92^ab^	70.71^b^	97.09^a^	97.48^a^	4.08	0.541	0.006	0.506
GR (U/g protein)	42.27	37.59	45.80	40.36	1.69	0.149	0.361	0.911
GSH (*μ*mol/mg wet weight)	0.45^a^	0.31^b^	0.47^a^	0.38^ab^	0.02	0.001	0.105	0.384
MDA (nmol/mg protein)	0.57^b^	0.90^a^	0.60^b^	0.56^b^	0.04	0.023	0.015	0.006
8-OHDG (ng/mg DNA)	0.85^b^	1.35^a^	0.75^b^	0.89^b^	0.07	0.017	0.034	0.158

^1^Data are presented as the means and SEM, *n* = 6. Mean values with different letters indicate a significant difference (*P* < 0.05). ^2^BW: birth weight; *B* × *D*: the interaction effect of the main effects (BW and diet); CAT: catalase; CON: basal diet; GSH: reduced glutathione; GSH-Px: glutathione peroxidase; GR: glutathione reductase; GST: glutathione S-transferase; IUGR: intrauterine growth retardation; MDA: malondialdehyde; NBW: normal birth weight; 8-OHDG: 8-hydroxy-2′-deoxyguanosine; PD: polydatin-supplemented diet; SOD: superoxide dismutase.

**Table 8:** Effect of dietary polydatin supplementation on the jejunal inflammatory responses of weanling piglets with different birth weights.

Items^1,2^	CON		PD		SEM	*P* value		
	NBW	IUGR	NBW	IUGR		BW	Diet	*B* × *D*
MPO (U/g protein)	41.58^b^	85.26^a^	46.71^b^	50.68^b^	5.31	0.010	0.096	0.029
TNF-*α* (pg/mg protein)	47.90^b^	94.52^a^	38.15^b^	47.00^b^	6.02	0.003	0.003	0.035
IL-10 (pg/mg protein)	5.09	5.06	6.05	5.32	0.43	0.680	0.509	0.700

^1^Data are presented as the means and SEM, *n* = 6. Mean values with different letters indicate a significant difference (*P* < 0.05). ^2^BW: birth weight; *B* × *D*: the interaction effect of the main effects (BW and diet); CON: basal diet; IL-10: interleukin-10; IUGR: intrauterine growth retardation; MPO: myeloperoxidase; NBW: normal birth weight; PD: polydatin-supplemented diet; TNF-*α*: tumor necrosis factor alpha.

**Table 9:** Effect of dietary polydatin supplementation on the jejunal energy metabolism of weanling piglets with different birth weights.

Items^1,2^	CON		PD		SEM	*P* value		
	NBW	IUGR	NBW	IUGR		BW	Diet	*B* × *D*
CS (U/mg protein)	15.11^a^	10.79^b^	17.36^a^	15.13^a^	0.78	0.021	0.020	0.432
Complex I (U/mg protein)	33.79^ab^	18.07^b^	41.31^a^	31.15^ab^	3.10	0.029	0.075	0.619
Complex II (U/mg protein)	19.38	19.42	13.34	16.97	1.47	0.540	0.165	0.549
Complex III (U/mg protein)	20.11^a^	7.65^b^	19.28^a^	19.88^a^	1.76	0.058	0.068	0.039
Complex IV (U/mg protein)	98.74	91.26	95.83	98.95	3.16	0.746	0.722	0.434
ATP synthase (U/mg protein)	19.69^a^	14.87^b^	17.64^ab^	21.54^a^	0.84	0.751	0.119	0.006
ATP (*μ*mol/g wet weight)	0.48^a^	0.33^b^	0.56^a^	0.49^a^	0.03	0.017	0.009	0.383

^1^Data are presented as the means and SEM, *n* = 6. Mean values with different letters indicate a significant difference (*P* < 0.05). ^2^ATP: adenosine triphosphate; BW: birth weight; *B* × *D*: the interaction effect of the main effects (BW and diet); CON: basal diet; CS: citrate synthase; IUGR: intrauterine growth retardation; NBW: normal birth weight; PD: polydatin-supplemented diet.

**Table 10:** Effect of dietary polydatin supplementation on the jejunal mitochondrial DNA content and sirtuin 1 activity of weanling piglets with different birth weights.

Items^1,2^	CON		PD		SEM	*P* value		
	NBW	IUGR	NBW	IUGR		BW	Diet	*B* × *D*
mtDNA (fold to NBW-CON)	1.00^a^	0.36^b^	0.91^a^	0.76^a^	0.07	0.003	0.211	0.048
SIRT1 (nmol/min/mg protein)	183.56^ab^	88.22^b^	284.02^a^	228.36^ab^	27.96	0.152	0.028	0.700

^1^Data are presented as the means and SEM, *n* = 6. Mean values with different letters indicate a significant difference (*P* < 0.05). ^2^ATP: adenosine triphosphate; BW: birth weight; *B* × *D*: the interaction effect of the main effects (BW and diet); CON: basal diet; CS: citrate synthase; IUGR: intrauterine growth retardation; NBW: normal birth weight; PD: polydatin-supplemented diet.

## Data Availability

The datasets used and analyzed during the current study are available from the corresponding author on reasonable request.

## Supplementary Materials

Supplementary Materials Supplementary Table 1: the composition and nutrient levels of diet.
