# Supplementary material for: Protective Effect of Polydatin on Jejunal Mucosal Integrity, Redox Status, Inflammatory Response, and Mitochondrial Function in Intrauterine Growth-Retarded Weanling Piglets
Source: Oxid Med Cell Longev. 2020 Oct 10;2020:7178123. doi: 10.1155/2020/7178123 (PMC7576365; doi:10.1155/2020/7178123)
Supplement: Supplementary Materials — Supplementary Table 1: the composition and nutrient levels of diet. [file 7178123.f1.docx]

**Supplementary Table 1.** Composition and nutrient levels of the diet

| Items | Contents |
| --- | --- |
| Ingredient (%) |  |
| Maize (8.7% CP) | 62.78 |
| Soybean meal (46.0% CP) | 15.00 |
| Fermented soybean meal (48.0% CP) | 7.00 |
| Extruded soybean (35.5% CP) | 7.00 |
| Soy protein isolate (86.2% CP) | 1.30 |
| Soyabean oil | 2.00 |
| CaHPO_4_ | 1.80 |
| Limestone | 0.80 |
| Salt | 0.35 |
| L-lysine-HCl (78.0%) | 0.52 |
| L-methionine | 0.13 |
| L-threonine | 0.15 |
| L-isoleucine | 0.10 |
| L-tryptophan | 0.01 |
| L-histidine | 0.01 |
| Calcium propionate (50.0%) | 0.05 |
| Premix^1^ | 1.00 |
| Total | 100.00 |
| Nutrient levels^2^ |  |
| Digestible energy (Mcal/kg) | 3.47 |
| Metabolizable energy (Mcal/kg) | 3.30 |
| Crude protein (%) | 20.36 |
| Total lysine (%) | 1.51 |
| Total methionine (%) | 0.46 |
| Total methionine + cystine (%) | 0.86 |
| Total threonine (%) | 0.94 |
| Total tryptophan (%) | 0.40 |
| Total histidine (%) | 0.77 |
| Total isoleucine (%) | 0.79 |
| Total valine (%) | 1.20 |
| Total calcium (%) | 0.82 |
| Total phosphorus (%) | 0.65 |

^1^Provide the following per kg complete diet: Vitamin A, 8,000 IU; Vitamin D_3_, 3,000 IU; Vitamin E, 20 IU; Vitamin K_3_, 3 mg; Vitamin B_1_, 2 mg; Vitamin B_2_, 5 mg; Vitamin B_6_, 7 mg; Vitamin B_12_, 0.02 mg; Niacin, 30 mg; Pantothenic acid, 15 mg; Folic acid, 0.3 mg; Biotin, 0.08 mg; Choline chloride, 500 mg; Fe (from ferrous sulfate), 110 mg; Cu (from copper sulfate), 7 mg; Mn (from manganese sulfate), 5 mg; Zn (from zinc sulfate), 110 mg; I (from calcium iodate), 0.3 mg; Se (from sodium selenite), 0.3 mg.

^2^All nutrient levels were analyzed values, except digestible energy and metabolizable energy.

^3^CP, crude protein.
